# Yield and Water Productivity Responses to Irrigation Cut-off Strategies after Fruit Set Using Stem Water Potential Thresholds in a Super-High Density Olive Orchard

**DOI:** 10.3389/fpls.2017.01280

**Published:** 2017-07-21

**Authors:** Luis E. Ahumada-Orellana, Samuel Ortega-Farías, Peter S. Searles, Jorge B. Retamales

**Affiliations:** ^1^Research and Extension Center for Irrigation and Agroclimatology, Facultad de Ciencias Agraria, Universidad de Talca Talca, Chile; ^2^Research Program on Adaptation of Agriculture to Climate Change (A2C2), Universidad de Talca Talca, Chile; ^3^Centro Regional de Investigaciones Científicas y Transferencia Tecnológica de La Rioja – Consejo Nacional de Investigaciones Científicas y Técnicas La Rioja, Argentina; ^4^Departamento de Horticultura, Facultad de Ciencias Agraria, Universidad de Talca Talca, Chile

**Keywords:** *Olea europaea*, deficit irrigation, plant water status, yield components, total oil yield

## Abstract

An increase in the land area dedicated to super-high density olive orchards has occurred in Chile in recent years. Such modern orchards have high irrigation requirements, and optimizing water use is a priority. Moreover, this region presents low water availability, which makes necessary to establish irrigation strategies to improve water productivity. An experiment was conducted during four consecutive growing seasons (2010–2011 to 2013–2014) to evaluate the responses of yield and water productivity to irrigation cut-off strategies. These strategies were applied after fruit set using midday stem water potential (Ψ_stem_) thresholds in a super-high density olive orchard (cv. Arbequina), located in the Pencahue Valley, Maule Region, Chile. The experimental design was completely randomized with four irrigation cut-off treatments based on the Ψ_stem_ thresholds and four replicate plots per treatment (five trees per plot). Similar to commercial growing conditions in our region, the Ψ_stem_ in the T_1_ treatment was maintained between -1.4 and -2.2 MPa (100% of actual evapotranspiration), while T_2_, T_3_ and T_4_ treatments did not receive irrigation from fruit set until they reached a Ψ_stem_ threshold of approximately -3.5, -5.0, and -6.0 MPa, respectively. Once the specific thresholds were reached, irrigation was restored and maintained as T_1_ in all treatments until fruits were harvested. Yield and its components were not significantly different between T_1_ and T_2_, but fruit yield and total oil yield, fruit weight, and fruit diameter were decreased by the T_3_ and T_4_ treatments. Moreover, yield showed a linear response with water stress integral (S_Ψ_), which was strongly influenced by fruit load. Total oil content (%) and pulp/stone ratio were not affected by the different irrigation strategies. Also, fruit and oil water productivities were significantly greater in T_1_ and T_2_ than in the T_3_ and T_4_. Moreover, the T_2_, T_3_, and T_4_ treatments averaged 37, 51, and 72 days without irrigation which represented 75–83, 62–76, and 56–70% of applied water compared with T_1_, respectively. These results suggest that using the T_2_ irrigation cut-off strategy could be applied in a super-high density olive orchard (cv. Arbequina) because it maintained yields, saving 20% of the applied water.

## Introduction

The olive tree (*Olea europaea* L.) is a characteristic species of the Mediterranean basin, which has traditionally been managed under dryland conditions. However, many studies have shown the benefits of irrigation on yield (Patumi et al., 2002; Moriana et al., 2003; Tognetti et al., 2007; Martín-Vertedor et al., 2011). For this reason, most of the commercial olive orchards in South America nowadays have been established at fairly high densities with drip-irrigation systems (Correa-Tedesco et al., 2010). Hedgerow orchards at super-high densities are also becoming a more common training system (Connor et al., 2014).

Despite yield gains at the farm level, increasing water scarcity in many regions has led to increased competition for water with non-agricultural users (Fereres et al., 2003). If less water is available, farmers should look toward increasing water productivity (production per unit of total water applied) through the optimization of irrigation management (Fereres and Evans, 2006; Iniesta et al., 2009; Fereres et al., 2014). For olive orchards, the regulated deficit irrigation (RDI) (Tognetti et al., 2005, 2007; Iniesta et al., 2009; Gómez del Campo and García, 2013) is the most commonly used irrigation strategy and consists of imposing water stress during phenological phases that are relatively insensitive to water deficit. Goldhamer (1999) reported that the pit hardening phase is the least sensitive to water deficit, and recommended the adoption of RDI, restricting irrigation during this phase. RDI strategies have achieved savings of around 20% of total water applied without reducing fruit yield (Goldhamer, 1999; Gómez-del-Campo, 2013). Additionally, these studies indicated that the oil content was not affected by the decrease in total amount of water applied. Moreover, Iniesta et al. (2009) observed that the water productivity for oil production was tripled when there was a 25% decrease in total applied water. Similarly, Correa-Tedesco et al. (2010) indicated that the greatest water productivity (21.3 kg mm^-1^ ha^-1^) was observed when applying water between 51 and 52% of actual evapotranspiration (ETc).

Traditionally, ETc is computed using grass reference evapotranspiration (ETo) multiplied by grass-reference-based crop-specific coefficients (Kc). ETo is estimated using the Penman–Monteith combination equation, but there is uncertainty on how to select the appropriate values of Kc. In this case, Kc values are empirical and often not adapted to local conditions (Ortega-Farias et al., 2009; Poblete-Echeverría and Ortega-Farías, 2013). The Kc in olive orchards depends on aspects of canopy architecture such as orientation (Connor et al., 2014), ground cover (Martínez-Cob and Faci, 2010), and the interactions of climatic conditions, soil type, cultivars and irrigation management practices (Ortega-Farías and López-Olivari, 2012). Due to this potential difficulty, recent research in olive trees has suggested using stem water potential (Ψ_stem_) to monitor plant water status and for scheduling water application (Moriana et al., 2012). Despite that some studies indicate that plant water status measurements could be strongly affected by the environment, which would question their usefulness as an irrigation scheduling tool (Corell et al., 2016), the water potential is a measurement commonly used as a reference in the description of water stress level (Moriana et al., 2012).

Irrigation cut-off strategies using water potential thresholds have been suggested for several researchers in prune, vineyards, and olive orchards (Lampinen et al., 2001; Girona et al., 2006; Moriana et al., 2012; Trentacoste et al., 2015). These strategies consist of suppressing irrigation completely during a given phenological phase which is insensitive to water deficit, and reestablishing irrigation only when a threshold value of Ψ_stem_ is reached. These strategies are easy to use for most farmers, since little knowledge is necessary about olive physiology in response to water stress.

In the literature, there is little information regarding irrigation strategies using Ψ_stem_ in olive trees. Moriana et al. (2012) observed that fruit yield decreased 30% in olive trees (cv. Cornicabra) that were irrigated when Ψ_stem_ fell below -2.0 MPa versus trees that were irrigated based on a -1.2 MPa threshold. Also, Fernandes-Silva et al. (2010) observed that olive trees (cv. Cobrancosa) irrigated when Ψ_stem_ reached -6.0 MPa had reductions greater than 50% in comparison with that of trees maintained under a Ψ_stem_ of around -3.0 MPa throughout the season. Ghrab et al. (2013) observed that the dry olive weight decreased significantly with Ψ_stem_ around -3.0 MPa. However, fruit yield for olive trees (cv. Frantoio) irrigated when the Ψ_stem_ dropped below -2.5 MPa, was statistically similar to the control (Ψ_stem_ threshold between -1.2 and -1.5 MPa) (Trentacoste et al., 2015). Correa-Tedesco et al. (2010) indicated that water deficit (Ψ_stem_ = -2.5 MPa) did not affect fruit weight. According to Dell’Amico et al. (2012), the lower yields can be attributed to the effect of water stress on fruit size. Finally, the effect of water deficit on yields depends on crop load, and is much more sensitive in years of high olive fruit load (Martín-Vertedor et al., 2011). This generates uncertainty regarding the use of Ψ_stem_ thresholds for irrigation in super-high density olive orchards (Naor et al., 2013). Due to these uncertainties, the objective of this study was to evaluate the yield and water productivity responses to irrigation cut-off strategies applied after fruit set using Ψ_stem_ thresholds in a super-high density olive orchard (cv. Arbequina).

## Materials and Methods

### Site Description and Experimental Design

The experiment was conducted during four consecutive growing seasons (2010–2011 to 2013–2014) in a 6-year-old drip-irrigated olive orchard (*O. europaea* L. cv. Arbequina), established in 2005 and located in the Pencahue Valley, Maule Region, Chile (35°, 232′ L.S; 71° 442′ W; 96 m altitude). The olive trees were trained under a hedgerow system with a planting density of 1333 tree ha^-1^ (1.5 × 5.0 m), and irrigated using two 2.0 L h^-1^ drippers per tree. The olive orchard was weekly irrigated from October to April based on ETc. The climate is Mediterranean with an annual rainfall of 620 mm, concentrated in the winter period (Ortega-Farías and López-Olivari, 2012). The soil texture is clay-loam (31% clay, 29% sand, and 40% silt), with a bulk density of 1.34 g cm^-3^, a field capacity of 0.31 cm^3^ cm^-3^, and a wilting point of 0.16 cm^3^ cm^-3^.

The irrigation requirements were calculated using the standard FAO56 formula for crop evapotranspiration (ETc = ETo × Kc) where ETo is the reference evapotranspiration estimated using the Penman–Monteith equation over grass (Ortega-Farías et al., 1995; Allen et al., 1998) and Kc is the crop coefficient. Climate data for determining ETo [temperature, relative humidity (RH), solar radiation, and wind speed] were obtained from an automatic meteorological station (AMS) installed at a reference grass area, located about 2 km SE from the experimental site. Moreover, effective rainfall (R) was calculated as R = (Pp - 5)^∗^0.75, where Pp = rainfall obtained from the AMS.

The experimental design was completely randomized with four treatments and four replications (five trees per replication). In treatment T_1_, the irrigation was calculated applying 100% of the ETc. In this case, crop coefficients (between 0.56 and 0.42) were obtained from López-Olivari et al. (2016). This treatment maintained a Ψ_stem_ value around -2.2 MPa during the months of maximum water demand. In other treatments, irrigation was cut-off from fruit set (20 days after full bloom) until reaching Ψ_stem_ thresholds of approximately -3.5 MPa in T_2_, -5.0 MPa in T_3_, and -6.0 MPa in T_4_ (Fernandes-Silva et al., 2010; Flores and Ortega-Farias, 2011). Once the specific thresholds were reached, the irrigation was reestablished in all treatments until fruits were harvested.

The phenological stages were determined according to the BBCH scale (Sanz-Cortes et al., 2002). In this scale, the pit hardening period was determined when the pit became lignified (shows resistance to cutting). Fernández et al. (2013) also call this period the maximum rate of pit hardening. The end-pit-hardening was determined when it was no longer possible to cut the fruit.

### Plant Water Status Measurements

The tree water status was monitored on a weekly basis using the midday stem water potential (Ψ_stem_). These measurements were performed between 12:30 and 14:00 h (midday solar time) (Moriana and Fereres, 2002; Gómez-Del-Campo et al., 2008) using two apical shoots per plot of the current year with at least 10 leaves, located in the middle zone of the canopy (Secchi et al., 2007; Rousseaux et al., 2008). These stems were covered with a plastic bag and aluminum foil for 1–2 h (Meyer and Reicosky, 1985) prior to measurements carried out using a Scholander-type pressure chamber (PMS Instrument Company, Model 1000 Pressure Chamber Instrument) (Scholander et al., 1965).

In order to describe the accumulated effect of the irrigation cut-off strategies, the water stress integral (*S*_Ψ_) was calculated as proposed by Myers (1988):

$$S_{\Psi }=\left|\sum \left({\bar{\Psi }}_{\text{stem}}\text{-}c\right)n\right|$$

where Ψ_stem_ is the average stem water potential for any interval (MPa), *c* is the value of the maximum stem water potential during the season, and *n* is the number of days in each interval (Moriana et al., 2007).

### Yield and Yield Components

To estimate fruit yield (kg ha^-1^), four trees from each plot were harvested manually on 130, 131, 134, and 127 DOY in 2011, 2012, 2013, and 2014, respectively. A sample of 50 olives from each replication was taken to measure their equatorial diameter as well as fruit weight, fresh pulp weight, and pulp/pit ratio using a precision balance. The total fruit number per tree was calculated by dividing the fruit yield of each tree by the individual fruit weight obtained previously (Patumi et al., 2002; Martín-Vertedor et al., 2011). Total oil content was determined following the official methods of AOAC using the Soxhlet method (Martín-Vertedor et al., 2011). This method extracted the oil by chemical methods and obtained all the lipids in the fruit. Total oil content was expressed on a dry weight basis (% d.w.). Water productivity was calculated as the ratio between fresh fruit yield (WP_f_) or total oil yield (WP_o_) per total water applied (irrigation + effective rainfall) during the growing season (Fernandes-Silva et al., 2013).

### Statistical Analysis

Treatment effects were evaluated by analysis of variance (ANOVA) using the statistical software Infostat (Universidad Nacional de Córdoba, Argentina). The significant differences among the treatments were assessed using Tukey’s multiple range test (*P* < 0.05). A regression analysis was performed to determine the relationship between water stress integral and fruit and oil yield.

## Results

### Environmental Conditions of the Study

**FIGURE 1:**
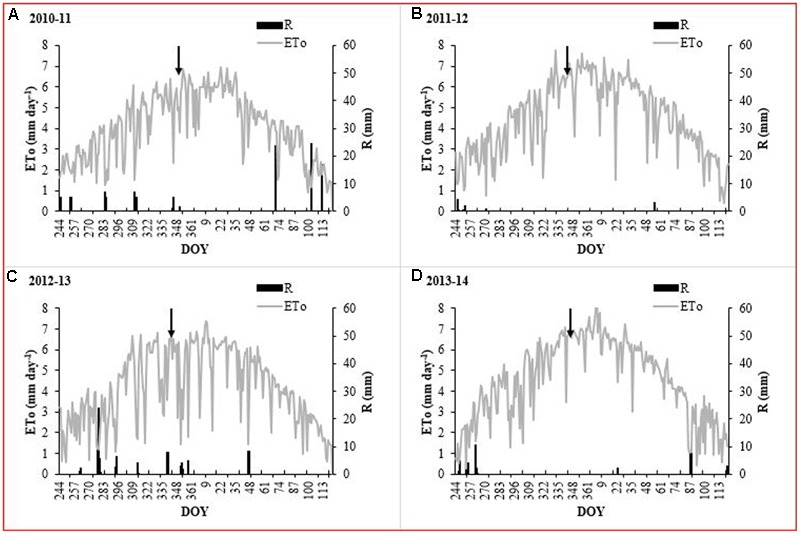
Reference evapotranspiration (ETo) and effective rainfall (R) during the 2010–2011, 2011–2012, 2012–2013 and 2013–2014 growing seasons (Pencahue Valley). The arrow indicates the beginning of the irrigation restriction of T_2_, T_3_ and T_4_.

**FIGURE 2:**
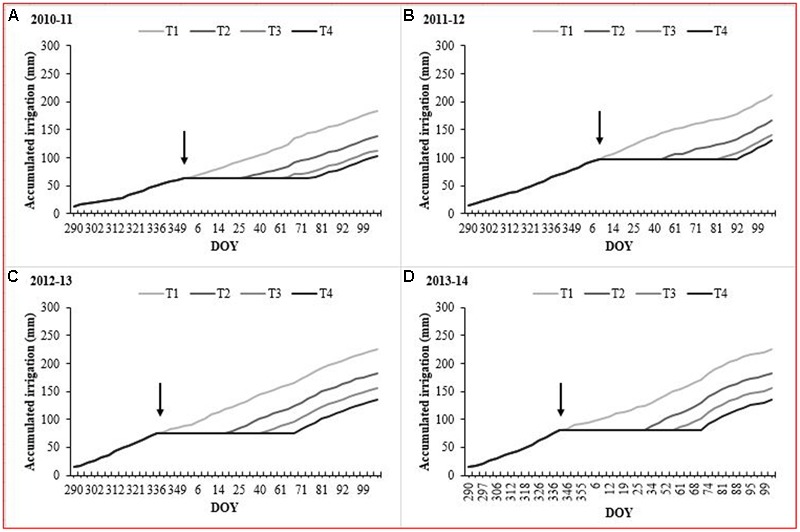
Accumulated irrigation (mm) of each treatments during the 2010–2011, 2011–2012, 2012–2013 and 2013–2014 growing seasons. The arrow indicates the beginning of the irrigation restriction of T_2_, T_3_ and T_4_.

The daily mean RH values at our experimental site ranged between 64.9 and 69.8%, while those of air temperature were between 15.7 and 16.5 °C for the four growing seasons (September to April) (**Table 1**). In addition, the 2013–2014 growing season had a higher thermal oscillation with maximum and minimum values of 26.8 and 5.9°C, respectively. The total reference ETo was between 986 and 1,099 mm for the four growth seasons (September to April). Maximum ETo was observed during December and January with values ranging between 5.4 and 6.6 mm day^-1^. The accumulated effective rainfall was 84.9, 12.6, 76.2, and 41.3 mm for the 2010–2011, 2011–2012, 2012–2013, and 2013–2014 growing seasons, respectively. However, in all seasons, accumulated rainfall was less than 30 mm during the water deficit period (December to March) (**Figure 1**). Under these atmospheric conditions, the irrigation during the 2010–2011 growing season was less than that of the following three seasons (**Figure 2**). In this experiment, irrigation for the T_2_, T_3_, and T_4_ was between 75 and 83, 62 and 76, and 56 and 70% of the T_1_ treatment, respectively (**Table 2**).

**Table 1:** Mean values of relative humidity (RH), air temperature (T), and reference evapotranspiration (ETo) during September and April.

Seasons	RH° (%)			T (°C)			ETo (mm season^-1^)
	Max.	Min.	Mean	Max.	Min.	Mean	
2010–2011	94.7	37.3	68.3	25.0	6.5	15.7	986
2011–2012	95.3	34.1	67.0	26.5	6.9	16.5	1094
2012–2013	96.0	36.5	69.8	26.2	6.5	16.0	1014
2013–2014	93.8	31.2	64.9	26.8	5.9	16.1	1099

*max., min., and mean are maximum, minimum, and mean values, respectively.*

**Table 2:** Irrigation and total water applied (mm ha^-1^) in each treatment during the 2010–2011, 2011–2012, 2012–2013, and 2013–2014 seasons.

Treatments	Irrigation				Total water applied^z^			
	2010–2011	2011–2012	2012–2013	2013–2014	2010–2011	2011–2012	2012–2013	2013–2014
T_1_	183.0	267.9	225.1	243.7	267.9	280.5	301.3	285.0
T_2_	137.6	222.5	182.4	195.7	222.6	235.2	258.6	237.0
T_3_	112.8	197.7	155.7	185.0	197.8	210.4	231.9	226.3
T_4_	102.3	187.2	134.4	156.3	187.3	199.9	210.6	197.6

*^z^Total water applied: Irrigation + effective rainfall.*

### Plant Water Status

**FIGURE 3:**
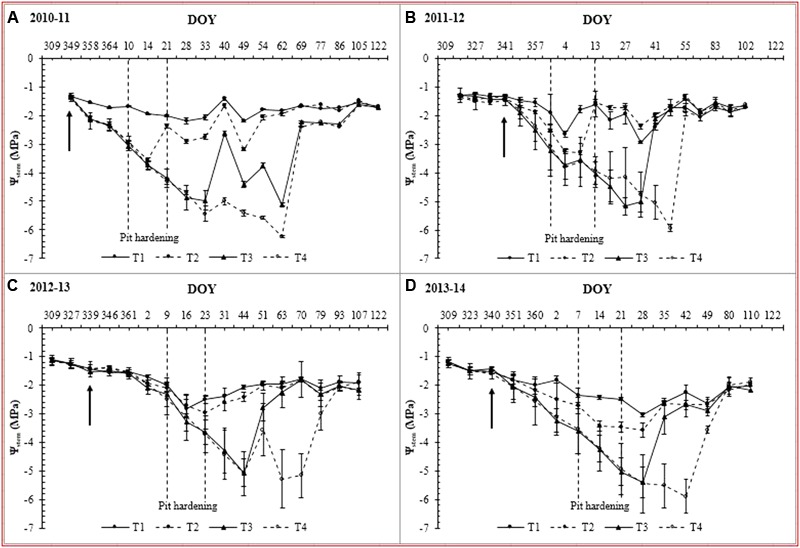
Evolution of midday stem water potential (Ψ_stem_) of each treatments during the 2010–2011, 2011–2012, 2012–2013 and 2013–2014 growing seasons. The dashed lines represent the beginning and end pit hardening (maximum rate of pit hardening). The arrow indicates the beginning of the irrigation restriction of T_2_, T_3_ and T_4_.

At fruit set (i.e., the start of water restriction), there were no significant differences among treatments for the tree water status, with Ψ_stem_ values ranging between -1.41 and -1.48 (**Table 3** and **Figure 3**). At the beginning of pit hardening (BPH), Ψ_stem_ values were lower in T_2_, T_3_, and T_4_ treatments than in the T_1_ treatment. Additionally, the T_3_ and T_4_ treatments had the lowest Ψ_stem_ at the BPH stage with -3.0 MPa, which were significantly lower than T_2_. At the end of pit hardening, on average there were no significant differences between the T_1_ and T_2_ treatments over the four growing seasons (**Table 3**) because the T_2_ treatments often reached the Ψ_stem_ threshold of -3.5 MPa (**Figure 3**), and the trees were re-watered (**Table 4**). The T_3_ and T_4_ treatments had lower values of Ψ_stem_ than the other treatments at the end of pit hardening because they had not reached their respective Ψ_stem_ thresholds (-5.0 and -6.0 MPa). The T_3_ treatment reached its Ψ_stem_ threshold between 49 and 53 days after the start of the irrigation cut-off, except for the 2012–2013 season (71 days; **Table 4**). The T_4_ treatment reached its threshold after 67–78 days for most years, but in the 2012–2013 season, it reached a minimum value of only -5.2 MPa after 97 days without irrigation. At harvest, values of Ψ_stem_ for all treatments ranged between -1.83 and -1.94 MPa with no significant differences among them. Finally, values of integral water stress (S_Ψ_) of T_1_ and T_2_ treatments were significantly lower than those of T_3_ and T_4_. The minimum and maximum S_Ψ_ values were 100.99 and 255.36 MPa, respectively (**Table 2**). T_4_ showed the highest S_Ψ_ of all treatments.

**Table 3:** Stem water potential (MPa) and water stress integral (S_Ψ_) for a drip-irrigation olive orchard at super-high density.

		Fruit set	Pit hardening (maximum rate)		Harvest	S_Ψ_ total (MPa day^-1^)
			Beginning	End		
Treatments					
	T_1_	-1.41	-1.98^a^	-2.16^a^	-1.84	100.99^c^
	T_2_	-1.42	-2.56^b^	-2.59^a^	-1.83	125.19^c^
	T_3_	-1.44	-3.05^c^	-4.25^b^	-1.94	210.10^b^
	T_4_	-1.48	-3.03^c^	-4.22^b^	-1.85	255.36^a^
Seasons					
	2010–2011	-1.35^a^	-2.66^b^	-3.22^a^	-1.71^a^	179.39^ab^
	2011–2012	-1.41^ab^	-2.68^b^	-2.81^a^	-1.68^a^	151.44^c^
	2012–2013	-1.46^bc^	-2.23^a^	-3.21^a^	-2.05^b^	161.47^bc^
	2013–2014	-1.52^c^	-3.05^b^	-3.98^b^	-2.02^b^	199.35^a^
ANOVA (*P-values*)					
Treatments	0.309	*<0.001*	*<0.001*	*0.582*	*<0.001*
Seasons	0.001	*<0.001*	*<0.001*	*<0.001*	*<0.001*

*Within each column data followed by different letters are significantly different according to the Tukey multiple comparison test (*P* < 0.05).*

**Table 4:** Days without irrigation for the irrigation cut-off strategies treatments and the stem water potential (Ψ_stem_) just before re-watering.

Seasons	Treatments	Days without irrigation	Ψ_stem_ (MPa)
2010–2011	T_2_	30	-3.55
	T_3_	49	-4.97
	T_4_	78	-6.23
2011–2012	T_2_	30	-3.29
	T_3_	51	-5.18
	T_4_	72	-5.94
2012–2013	T_2_	50	-2.98
	T_3_	71	-5.09
	T_4_	97	-5.17
2013–2014	T_2_	39	-3.43
	T_3_	53	-5.40
	T_4_	67	-5.89

### Yield and Yield Components

Average fruit yield in the four seasons was not significantly different between T_1_ and T_2_, with a yield of 11,984 and 10,917 kg ha^-1^, respectively (**Table 5**). However, both treatments had fruit yields significantly greater than those of the T_3_ and T_4_ treatments. Average crop load was greater in T_2_ (7,966 olives tree^-1^) compared to T_3_ and T_4_, but there was no significant difference between T_1_ and T_2_.

**Table 5:** Fruit yield and its components for each treatment and growing season.

		Fruit yield (kg ha^-1^)	Crop load (Fruit tree^-1^)	Equa. diameter (mm)	Fresh fruit weight (g)	Fresh pulp weight (g)	Pulp:Pit ratio
Treatments						
	T_1_	11,984^a^	7,211^ab^	11.83^a^	1.29^a^	0.95^a^	2.97
	T_2_	10,917^a^	7,966^a^	11.39^b^	1.10^b^	0.84^b^	3.20
	T_3_	7,998^b^	6,522^b^	10.91^c^	0.97^c^	0.74^c^	3.36
	T_4_	7,305^b^	6,309^b^	10.55^c^	0.91^c^	0.69^c^	3.31
Seasons						
	2010–2011	11,637^a^	10,737^a^	10.15^c^	0.81^c^	0.61^b^	3.24^b^
	2011–2012	9,411^b^	6,371^b^	11.29^b^	1.10^b^	0.87^ab^	3.85^a^
	2012–2013	7,811^c^	5,225^c^	11.53^ab^	1.14^ab^	0.83^a^	2.79^b^
	2013–2014	9,344^b^	5,675^bc^	11.71^a^	1.22^a^	0.90^a^	2.96^b^
ANOVA (*P-values*)						
Treatments	*<0.001*	*0.004*	*<0.001*	*<0.001*	*<0.001*	0.175
Seasons	*<0.001*	*<0.001*	*<0.001*	*<0.001*	*<0.001*	*<0.001*

*Within each column data followed by different letters are significantly different according to the Tukey multiple comparison test (*P* < 0.05).*

Yield components including equatorial diameter, fresh fruit weight, and fresh pulp were all affected by the irrigation cut-offs strategies (**Table 5**). They had their highest values in the T_1_ treatment and decreased progressively with increased water deficit, reaching their lowest values in T_3_ and T_4_. The pulp/pit ratio did not show significant differences among treatments with values ranging between 2.97 and 3.36.

Furthermore, there were significant differences between seasons for yield and its components. Fruit yield was greatest in the 2010–2011 season and lowest in the 2012–2013 season. Crop load had a similar pattern to yield, with the greatest crop load observed in the 2010–2011 season and the lowest crop loads occurring in the 2012–2013 and 2013–2014 seasons. The fruit equatorial diameter and individual fruit fresh weight were lower in the season with the highest crop load (2010–2011) and greater when the crop load was low (2012–2013 and 2013–2014 seasons). Fresh pulp weight was significantly less in the 2010–2011 season than in the other three seasons, and pulp/pit ratio was significantly higher in the 2011–2012 season.

### Total Oil Content and Water Productivity

The average total oil content of treatments in the four seasons was between 46.7 and 50.2% (d.w.), but there were no significant differences among them (**Table 6**). Total oil yield reflected the fruit yield pattern, with the total oil yield being greater in the T_1_ and T_2_ treatments (2439 and 2199 kg ha^-1^, respectively) than in the T_3_ and T_4_ treatments (1,560 and 1,440 kg tree^-1^, respectively). The fruit (WP_f_) and oil (WP_o_) water productivities were significantly greater in the T_1_ and T_2_ than in the T_3_ and T_4_ treatments. It is important to indicate that WP_o_ was calculated using total oil content obtained as fruit yield multiplied by % oil obtained from Soxhlet method (Fernandes-Silva et al., 2013).

**Table 6:** Total oil content, oil yield, and fruit (WP_f_) and oil (WP_o_) water productivity for each treatment and growing season.

		Total oil content (bdw %)	Total oil yield (kg ha^-1^)	WP_f_ (kg m^-3^)	WP_o_ (kg m^-3^)
Treatments				
	T_1_	50.19	2,439^a^	42.6^a^	8.63^a^
	T_2_	48.62	2,199^a^	46.4^a^	9.33^a^
	T_3_	47.04	1,560^b^	37.3^b^	7.34^b^
	T_4_	46.72	1,440^b^	36.8^b^	7.36^b^
Seasons				
	2010–2011	51.70^b^	2,119^a^	52.5^a^	9.63^a^
	2011–2012	56.48^a^	2,333^a^	40.4^b^	9.96^a^
	2012–2013	47.31^c^	1,653^b^	31.4^c^	6.70^b^
	2013–2014	37.10^d^	1,533^b^	38.7^b^	6.36^b^
ANOVA (*P-values*)				
Treatments	*0.0052*	*<0.001*	*<0.001*	*<0.001*
Seasons	*<0.001*	*<0.001*	*<0.001*	*<0.001*

*Within each column data followed by different letters are significantly different according to the Tukey multiple comparison test (*P* < 0.05).*

The highest total oil content occurred in the 2011–2012 season with an average of 56.5% and the lowest total oil content in the 2013–2014 season with 37.2%, while the total oil yield was significantly greater in 2010–2011 and 2011–2012 than in 2012–2013 and 2013–2014. The greatest WP_f_ was found in the 2010–2011 season with 52.5 kg fruit mm^-1^ and the lowest was in the 2012–2013 season with 31.4 kg fruit mm^-1^. For WP_o_, the maximum values were observed in 2010–2011 and 2011–2012 (9.63 and 9.96 kg oil mm^-1^, respectively).

## Discussion

Our region has a cool Mediterranean-type climate with about 620 mm of rainfall occurring during the winter months. During this study, weather behaved according to the expected conditions in the area, with maximum atmospheric demand during December and January. Due to low rainfall in summer, we found that irrigation was necessary to maintain fruit yield and total oil yield, and that irrigation cut-offs based on Ψ_stem_ thresholds was a practical and user-friendly method of scheduling irrigation in super-high density orchards. However, we recognize that optimal midday stem water potential may vary somewhat by region due to climate variables such as vapor pressure deficit and temperature (Corell et al., 2016), or due to soil type.

Irrigation of the T_1_ treatment averaged 230 mm over the four growing seasons. For these seasons, Ψ_stem_ mostly ranged between -1.4 and -2.0 MPa, indicating that trees were in a null to mild water stress condition. This can be established because despite that thresholds from -1.0 to -1.5 MPa have been suggested as adequate to satisfy olive tree water requirements (Dell’Amico et al., 2012), values lower than -2.0 MPa can occur even in well-watered trees during the summer under high vapor pressure deficits and high crop load conditions (Ben-Gal et al., 2010; Ortega-Farías and López-Olivari, 2012; Marra et al., 2016). The T_2_ treatment received an average of 185 mm per growing season, which was almost 20% less than the T_1_ treatment. This treatment reached its Ψ_stem_ threshold (-3.5 MPa) during pit hardening after an average of 37 days without irrigation (**Table 4**). Reducing irrigation during pit hardening has been recommended by many authors because this phase is the least sensitive to water deficit (Goldhamer, 1999; Alegre et al., 2002; Gómez del Campo and García, 2013). Also, the application of RDI during this phase could allow for considerable water savings because pit hardening generally coincides with high atmospheric demands for water vapor (Goldhamer, 1999). In our study, the pit hardening period (maximum rate) was reached during January, which coincides with the time of the year observed in previous seasons (López-Olivari et al., 2016). The T_3_ and T_4_ treatments received an average of 163 and 141 mm of irrigation per growing season, respectively, and reached their Ψ_stem_ thresholds post-pit hardening (post-maximum rate) after 56 and 72 days without irrigation. These thresholds (-5.0 MPa in T_3_ and -6.0 MPa in T_4_) suggest that the trees in the T_3_ and T_4_ treatments were severely stressed during the experiment (Moriana et al., 2002). Once irrigation was restored in the T_2_, T_3_, and T_4_ treatments, their Ψ_stem_ returned to values similar to those of the T_1_ treatment as has been observed by other authors (Dell’Amico et al., 2012; Agüero et al., 2016).

The orchard was severely pruned during the spring of the 2012–2013 season, which likely decreased the daily crop water requirements due to reduced leaf area per tree. This lower demand could explain the extended period without irrigation in the T_2_ and T_3_ treatments before reaching their Ψ_stem_ threshold in 2012–2013 (50 and 71 days in T_2_ and T_3_, respectively). In the case of the T_4_ treatment, the combination of spring pruning and rainfall in December (23.9 mm) resulted in the Ψ_stem_ threshold not being reached in the 2012–2013 season.

Despite receiving almost 20% less irrigation, fruit yield of the T_2_ treatment (10,917 kg ha^-1^) was not statistically lower than that of the T_1_ treatment (11,984 kg ha^-1^) for the four growing seasons. This suggests that this irrigation cut-off strategy could be applied in commercial orchards without affecting yield. In contrast, the fruit size was reduced by 15% (T_2_), although maximum water stress occurred after the vast majority of endocarp (pit) and mesocarp (pulp) cells were formed (Hammami et al., 2011). This reduction can be explained because fruit expansion requires an adequate flow of water to the fruit and sufficient turgor to drive in cell enlargement (Dell’Amico et al., 2012). These results are in accordance with Marra et al. (2016), who suggested maintaining Ψ_stem_ values between -3.5 and -2.5 MPa to get an optimal-moderate yield in olive cv. Arbequina.

Fruit yields for T_3_ and T_4_ treatments were 33 and 39% less than T_1_, respectively. These reductions were mainly due to a smaller crop load and lower fruit weight as has been reported by Fernandes-Silva et al. (2010) for severe water stress conditions. Crop load was reduced by 10–13% in T_3_ and T_4_ treatments, respectively, and fruit weight was reduced by 25–30%. This decrease in yield and their components can be explained by limitations to photosynthesis which are controlled by water stress (Angelopoulos et al., 1996; Flexas and Medrano, 2002). Indeed, the immediate response of plants to water stress is to limit leaf transpiration in order to reduce water loss through stomatal closure (Fernández et al., 1997). However, this also causes reduced CO_2_ diffusion into the leaf, thereby limiting carbon assimilation (Angelopoulos et al., 1996; Ennajeh et al., 2008).

**FIGURE 4:**
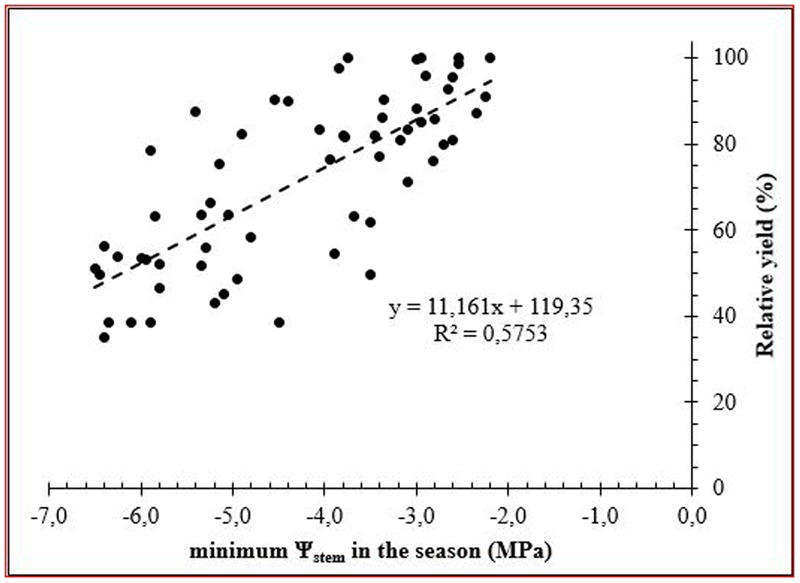
Relationship between minimum values of midday stem water potential (Ψ_stem_) and relative yield during the study seasons.

**FIGURE 5:**
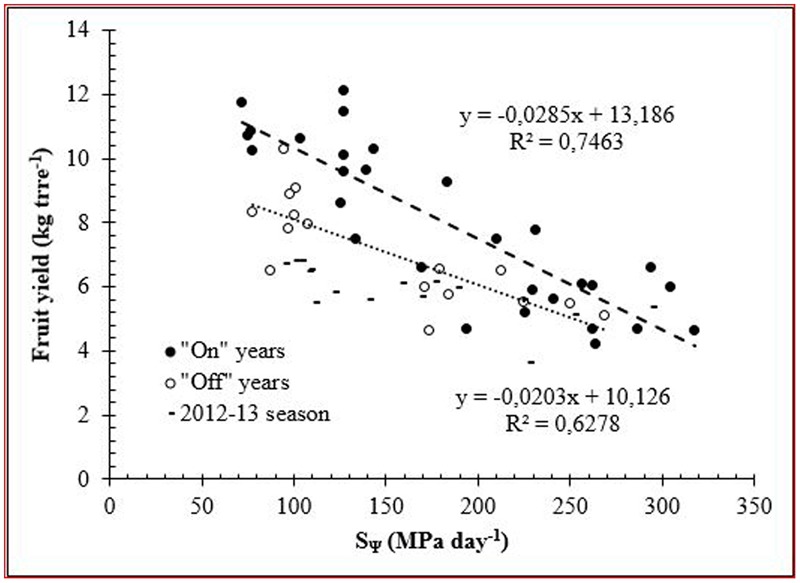
Relationship between integral water stress (S_Ψ_) and fruit yield during the study seasons. On years: 2010–2011 and 2013–2014. Off years: 2011–2012.

Moreover, relative fruit yield showed a linear response with the minimum Ψ_stem_ during the seasons, despite that there was a high dispersion of data (**Figure 4**). However, an integrated plant water status is more appropriated for evaluating the effect of water deficit on fruit yield. Therefore, the S_Ψ_ was related to fruit yield and had a high linear correlation (**Figure 5**). These results coincide with those reported by Moriana et al. (2012) in olive trees cv. Cornicabra. However, in our results, this relationship was strongly influenced by fruit load. Thus, in the “on” years, the relationship was higher and presented a greater slope than in “off” years.

Fruit yields of all treatments followed a biannual (alternate bearing) pattern with an “on” year in 2010–2011 and an “off” year in 2011–2012. Unfortunately, the pruning done in the 2012–2013 season prevented the assessment of alternate bearing in the last two seasons. Such a pattern is common in most olive cultivars (Lavee, 2007). This often occurs because in the “on” year there is low shoot growth, which leads to few potentially reproductive buds for the next year; when crop load is high there is inhibition of floral induction (Dag et al., 2010; Fernández et al., 2015). Consequently, the year-to-year variations in yield are directly related with fruit number per tree of each year (Martín-Vertedor et al., 2011; Trentacoste et al., 2015). Additionally, fruit size and fruit weight were lower in the seasons with higher crop loads. These results are explained by the close relationship between yield components and the number of olives per tree (Iniesta et al., 2009; Martín-Vertedor et al., 2011).

Water deficit treatments did not lead to any changes in total oil content (%). Therefore, the reductions in oil yield in the T_3_ and T_4_ treatments were related to crop load and fruit weight, rather than to total oil content itself. Total oil content on a dry weight basis appears to be fairly insensitive to water deficit (Patumi et al., 2002; Moriana et al., 2003; Tognetti et al., 2007; Iniesta et al., 2009; García et al., 2013). However, Trentacoste et al. (2015) found a significant reduction of 3.1% in total oil content using a -2.5 MPa irrigation threshold in cv. Frantoio. The lack of response of oil content (%) in many of these studies is likely a function of water deficit being implemented during the pit hardening period, rather than later when most oil accumulation occurs. Moreover, total oil content was significantly lower in the 2010–2011 season compared to other seasons. In this case, air temperature (minimum and maximum) and maturity index (MI) were lower during April 2011 (40 days before harvest) than in other years. In this case, the MI were 1.56, 1.69, 1.56, and 1.11 for 2010–2011, 2011–2012, 2012–2013, and 2013–2014, respectively. These results coincide with those observed by Motilva et al. (2000) and Grattan et al. (2006) who suggest that total oil content is highly related to MI.

Despite T_2_ receiving almost 20% less irrigation, no significant differences in WP_f_ and WP_o_ were found between the T_1_ and T_2_ treatments. In contrast, WP decreased for both fruit and oil yield under severe water stress in the T_3_ and T_4_ treatments. Thus, the WP responses would be explained by the difference in the intensity of water deficit treatments. The decrease in WP_f_ and WP_o_ under severe stress is consistent with the results of Fernandes-Silva et al. (2010) in olive cv Cobrançosa where water potential also reached around -6.0 MPa. Some studies have reported an increase in WP_f_ under moderate stress conditions with Ψ_stem_ values similar to those observed with the T_2_ treatment (Marra et al., 2016). It may be that an intermediate Ψ_stem_ threshold between our T_2_ and the T_3_ and T_4_ treatments may have led to a significant increase in WP_f_ and WP_o_.

## Conclusion

Results obtained in the present study over four growing seasons showed that yields were not affected when irrigation cut-off was applied from fruit set until reaching a threshold level of -3.5 MPa (T_2_ treatment) around massive pit hardening compared to T_1_ (100% ETc). This provides evidence that this period is not overly sensitive to moderate water stress. However, yield and its components were severely affected when using Ψ_stem_ thresholds of -5.0 (T_3_) and -6.0 (T_4_) MPa. Also, the total oil content (%) and pulp/pit ratio were not affected by the different irrigation cut-off strategies. Moreover, the fruit and oil water productivities were significantly greater in T_2_ compared to T_3_ and T_4_ treatments.

In summary, these results suggest that the T_2_ irrigation cut-off strategy would be the most appropriate, because this treatment maintained fruit and oil yield, saving 20% of the total water applied. These results suggest that this strategy (T_2_) is a viable strategy to be implemented in high-density olive orchards in climates similar to the one where this research was done.

## Author Contributions

Conceived and designed the experiments: SO-F and LA-O. Performed the evaluations: LA-O. Analyzed the data: SO-F, LA-O, PS, and JR. Wrote the paper: LA-O, SO-F, PS, and JR. Implemented reviewers comments: SO-F, LA-O, PS, and JR.

## Conflict of Interest Statement

The authors declare that the research was conducted in the absence of any commercial or financial relationships that could be construed as a potential conflict of interest.
